# Mechanical stretch leads to increased caveolin-1 content and mineralization potential in extracellular vesicles from vascular smooth muscle cells

**DOI:** 10.1186/s12860-024-00504-w

**Published:** 2024-03-14

**Authors:** Mohammad Shaver, Kassandra Gomez, Katherine Kaiser, Joshua D. Hutcheson

**Affiliations:** 1 Department of Biomedical Engineering, Florida International University, 10555 West Flagler Street, Engineering Center 2600, Miami, FL 33174 USA; 2 Biomolecular Sciences Institute, Florida International University, Miami, FL 33199 USA

**Keywords:** Mechanical stretch, Extracellular vesicles, Caveolin-1, Calcification, Vascular smooth muscle cells

## Abstract

**Background:**

Hypertension-induced mechanical stress on vascular smooth muscle cells (VSMCs) is a known risk factor for vascular remodeling, including vascular calcification. Caveolin-1 (Cav-1), an integral structural component of plasma membrane invaginations, is a mechanosensitive protein that is required for the formation of calcifying extracellular vesicles (EVs). However, the role of mechanics in Cav-1-induced EV formation from VSMCs has not been reported.

**Results:**

Exposure of VSMCs to 10% mechanical stretch (0.5 Hz) for 72 h resulted in Cav-1 translocation into non-caveolar regions of the plasma membrane and subsequent redistribution of Cav-1 from the VSMCs into EVs. Inhibition of Rho-A kinase (ROCK) in mechanically-stimulated VSMCs exacerbated the liberation of Cav-1 positive EVs from the cells, suggesting a potential involvement of actin stress fibers in this process. The mineralization potential of EVs was measured by incubating the EVs in a high phosphate solution and measuring light scattered by the minerals at 340 nm. EVs released from stretched VSMCs showed higher mineralization potential than the EVs released from non-stretched VSMCs. Culturing VSMCs in pro-calcific media and exposure to mechanical stretch increased tissue non-specific alkaline phosphatase (ALP), an important enzyme in vascular calcification, activity in EVs released from the cells, with cyclic stretch further elevating EV ALP activity compared to non-stretched cells.

**Conclusion:**

Our data demonstrate that mechanical stretch alters Cav-1 trafficking and EV release, and the released EVs have elevated mineralization potential.

**Supplementary Information:**

The online version contains supplementary material available at 10.1186/s12860-024-00504-w.

## Introduction

Vascular calcification results from bone-like mineral formation in the arterial wall and correlates significantly with cardiovascular morbidity and mortality [1]. For decades, calcification was considered a degenerative process due to aging; however, growing evidence has shown that calcification occurs in an actively regulated process similar to bone remodeling [2]. Calcification often begins in specialized extracellular vesicles (EVs) released from vascular smooth muscle cells (VSMCs) in response to pathological stimuli [3]. Once released, these EVs sequester calcium and phosphate ions to nucleate mineral formation [4]. Aggregation of the EVs leads to mineral growth, which disrupts the biomechanical function of the arterial wall and can directly contribute to both acute and chronic cardiovascular pathologies [5, 6].

Goettsch et al. reported that the formation of calcifying EVs requires the presence of caveolin-1 (Cav-1), a structural protein important in the formation of plasma membrane invaginations known as caveolae [7]. These 50–100 nm flask shape invaginations in the plasma membrane contain abundant cholesterol, sphingolipids, and scaffolding proteins [8–11]. Caveolin-1 is the major membrane protein in the caveolae structure and is involved in endocytosis, lipid trafficking, cell signaling, EV biogenesis, and mechanotransduction [12, 13]. Caveolae, in association with actin filaments, respond to mechanical stimulation such as membrane stretch [12, 14]. Increased tension on the cell membrane leads to caveolae destabilization and disassembly [15]. Plasma membrane deformation is associated with rapid cytoskeletal reorganization, which leads to caveolae endocytosis [8]. Caveolin-1 internalization and trafficking through the Golgi apparatus, begins the formation of calcifying EVs [7, 16]. However, no studies have been conducted to investigate the role of mechanics in the generation of calcifying EVs.

Hypertension, a major risk factor for cardiovascular diseases, abnormally increases mechanical stretch on the arterial wall [17, 18]. The main responders to mechanical stretch are VSMCs, which are abundant in the arterial wall and responsible for the maintenance of acute vascular homeostasis through active contraction and relaxation [19–21]. Sustained pathological stressors cause VSMCs to adopt a synthetic phenotype associated with increased expression of osteogenic proteins and deposition of mineralized bone-like matrix [22]. Calcification leads to arterial remodeling and alters arterial mechanics in a way that reduces elasticity and increases the stiffness [23]. In vitro studies have modeled vascular calcification by either culturing VSMCs in media designed to promote osteogenic alterations in phenotype [24] or in mineralization-competent media that alter phosphate homeostasis [23]. Studies into the effects of mechanical stimulation on caveolin-1 trafficking and EV formation could yield new insight into how altered mechanics affects vascular remodeling and calcification in response to these pathological cues.

In order to examine the interrelation between mechanical stimulation, caveolin-1-dependent EVs formation, and the propensity of these vesicles to induce calcification, VSMCs were cultured in a cyclic strain environment in vitro. First, we show that mechanical stretch redistributes caveolin-1 from VSMCs into EVs. Measurements of calcification potential of the released EVs demonstrated enhanced mineralization competency in EVs from stretched VSMCs.

## Results

### Mechanical stretch induces caveolin-1 translocation within plasma membrane of VSMCs

**Fig. 1:**
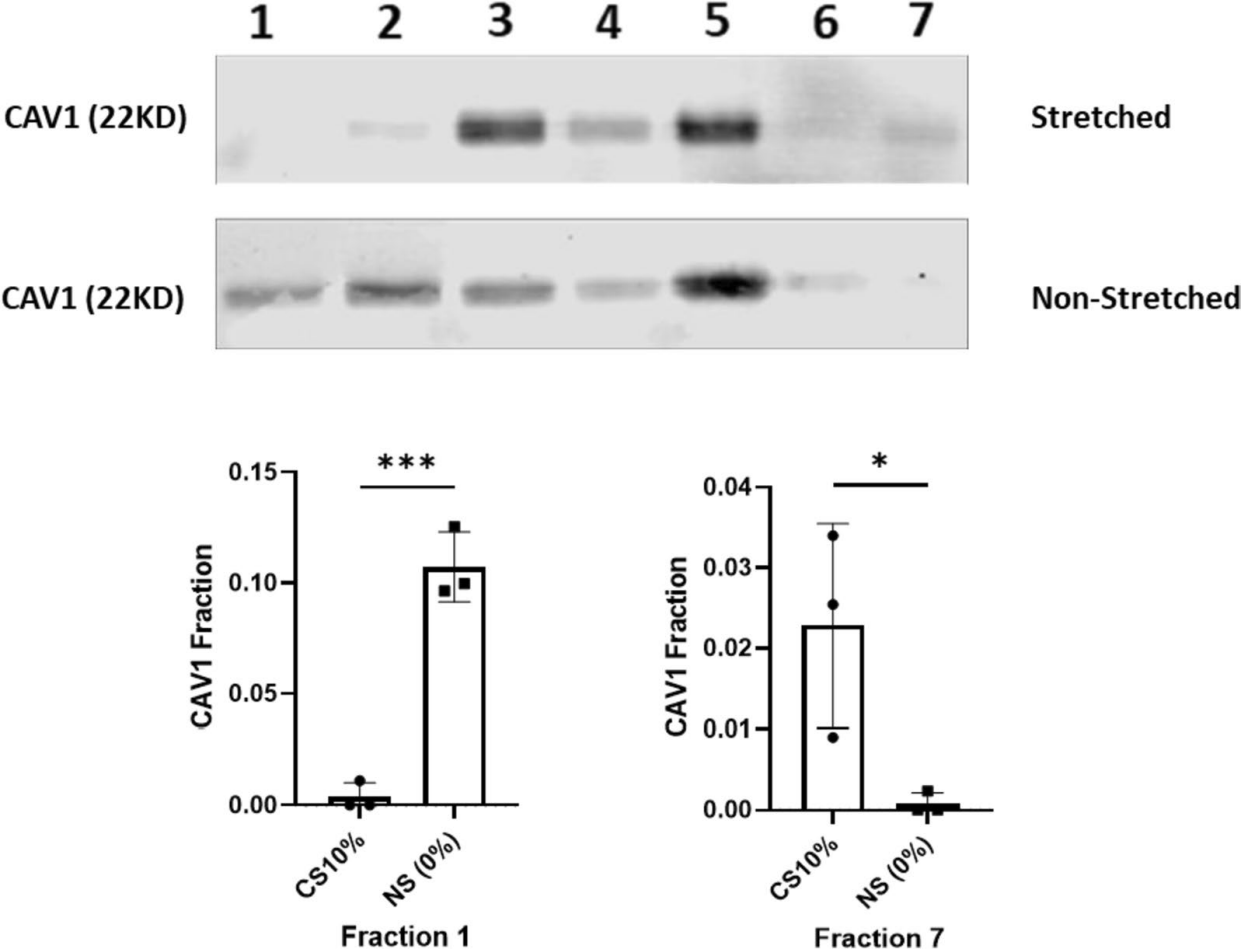
Effect of mechanical strain on subcellular localization of caveolin-1 in vascular smooth muscle cells (VSMCs). **A** Representative western blots showing caveolin-1 (Cav-1) protein levels in caveolar (fraction 1) and non-caveolar (fraction 7) membrane fractions isolated from non-stretched VSMCs and VSMCs exposed to 10% cyclic stretch for 72 h. **B** Densitometry analysis of Cav-1 revealed that cyclic stretch induced a significant translocation of Cav-1 from the caveolar fraction to the non-caveolar fraction compared to non-stretched control cells *n* = 3 (*P < 0.05, ***P < 0.001). Data represents mean ± SD. Full-length blots are presented in Supplementary Fig. 1

Due to their unique lipid and protein composition, caveolae exhibit a low buoyant density enabling separation from other cellular fractions through gradient centrifugation [25]. To investigate whether mechanical stretch alters the subcellular localization of Cav-1 within VSMC plasma membranes, caveolar and non-caveolar membrane fractions were separated by discontinuous sucrose gradient ultracentrifugation. Immunoblotting analysis revealed that in non-stretched control VSMCs, Cav-1 was detected in fractions 1–5. Following 72 h of cyclic stretch, Cav-1 displayed a clear shift in localization to more dense membrane fractions (Fig. 1). These data demonstrate that prolonged mechanical stretch induces the translocation of Cav-1 from caveolae domains to non-caveolar regions of the plasma membrane in VSMCs.

### Mechanical stretch redistributes caveolin-1 from VSMCs into EVs

**Fig. 2:**
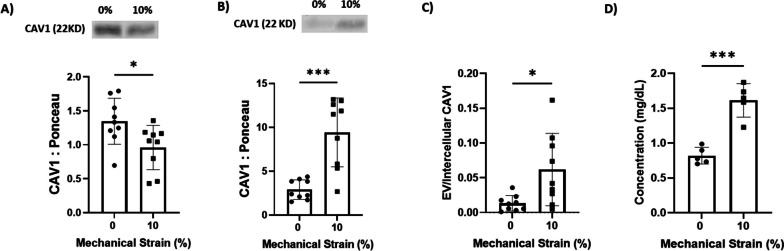
Effect of cyclic strain on caveolin-1 distribution in vascular smooth muscle cells (VSMCs). **A** Application of 10% cyclic strain for 72 h significantly decreases the intracellular level of caveolin-1 compared to non-stretched control VSMCs, as quantified by Western blotting. **B** Exposure of VSMCs to 10% cyclic strain for 72 h significantly increases the release of caveolin-1 positive extracellular vesicles (EVs) compared to non-stretched controls, as determined by Western blotting. **C** Cyclic strain results in increased trafficking of caveolin-1 from VSMCs into extracellular vesicles as compared to static control conditions, based on normalization of extracellular vesicular caveolin-1 content to intracellular caveolin-1 levels in corresponding VSMCs. **D** Cyclic mechanical strain elevates intracellular cholesterol levels in VSMCs. *n* = 9 Data are presented as mean ± SD (*P < 0.05, ****P < 0.0001). Data represents mean ± SD. Full-length blots are presented in Supplementary Fig. 2

Next, we investigated the role of 72 h of cyclic mechanical stimulation (0.5 Hz) in Cav-1 trafficking and EV release in VSMCs. Quantitative analysis of Western blot data demonstrated a statistically significant 1.39-fold reduction (*P* < 0.01) in cellular Cav-1 protein levels in mechanically stimulated VSMCs compared to static control cells (Fig. 2A). In contrast, Cav-1 was increased by 3.41-fold (*P* < 0.0001) in EVs secreted from VSMCs exposed to 10% cyclic stretch versus EVs from non-stretched controls (Fig. 2B). Normalizing the Cav-1 content in EVs to the intracellular levels in corresponding VSMCs revealed a 4.77-fold (*P* < 0.0001) higher EV-to-VSMC Cav-1 ratio in the 10% cyclic stretch condition compared to the static condition (Fig. 2C). Quantification of total intracellular cholesterol levels revealed that application of cyclic mechanical stretch for 24 h elicited a significant 1.96-fold (*P* < 0.001) increase in cholesterol abundance within VSMCs compared to non-stretch control cells (Fig. 2D).

### Mechanical stretch leads to caveolin-1 internalization

**Fig. 3:**
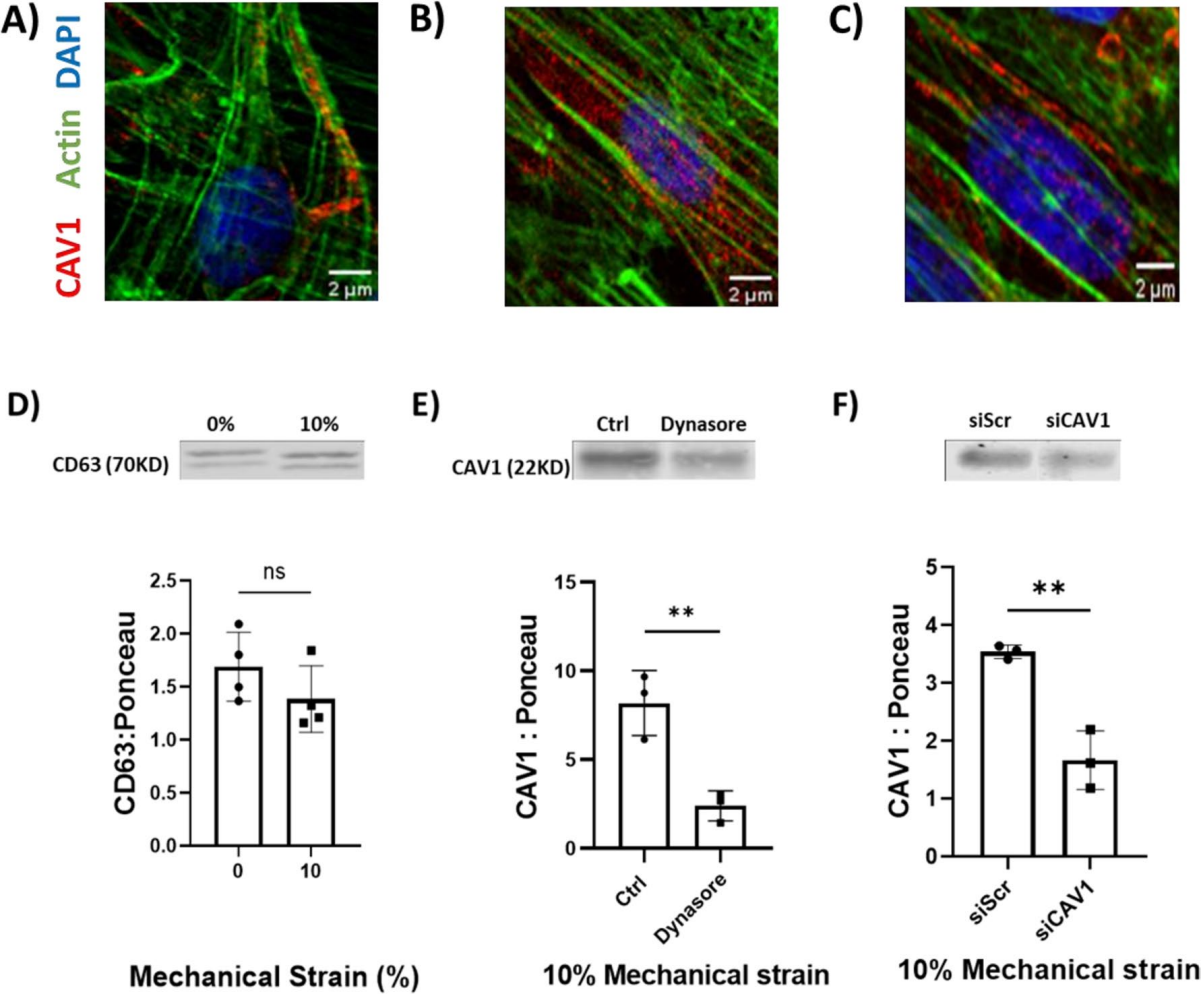
Immunofluorescence staining shows Cav-1 (red) transitioning from alignment along actin filaments in non-stretched VSMCs)(**A**) to a more dispersed, cytosolic distribution after application of 10% cyclic strain for 72 h (**B**). **C** Pre-treatment with the dynamin inhibitor dynasore (40 μM) reduces Cav-1 internalization in VSMCs subjected to 10% strain for 72 h compared to control. **D** This inhibition of Cav-1 endocytosis by dynasore decreases Cav-1 release in EVs from stretched VSMCs by 2.41-fold (*P* < 0.01). **E** Levels of the exosomal marker CD63 are unchanged in EVs from control versus 10% stretched VSMCs. **F** Knockdown of the Cav-1 gene in cyclically stretched VSMCs significantly decreased Cav-1 protein levels in EVs released from these cells. *n* = 3. Data are mean ± SD (**P < 0.01) (Scale bar: 2 µm). Data represents mean ± SD. Full-length blots are presented in Supplementary Fig. 3

Immunofluorescence imaging revealed changes in Cav-1 localization within VSMCs after application of mechanical stretch. In non-stretched VSMCs, Cav-1 predominantly aligned along actin filaments (Fig. 3A), while application of 10% equibiaxial cyclic stretch for 72 h induced a redistribution of Cav-1 from actin-associated regions to more dispersed regions within the cytosol (Fig. 3B). To investigate if Cav-1 mobilization in response to mechanical factors involved dynamin-dependent endocytosis, VSMCs were treated with the dynamin inhibitor dynasore prior to 10% stretch for 72 h. Inhibition of dynamin activity resulted in the retention of Cav-1 along actin filaments (Fig. 3C), similar to the pattern observed in static VSMCs. This reduction in Cav-1 internalization upon dynasore treatment was accompanied by a 3.41-fold (*P* < 0.01) decrease in the levels of Cav-1 released extracellular vesicles (Fig. 3D). Moreover, we did not observe any significant change in the value of exosome marker CD63 in the isolated EVs of 10% stretched VSMCs compared to non-stretched cells after 72 h (Fig. 3E). Quantitative immunoblotting revealed a statistically significant 2.21-fold (*P* < 0.01) reduction in Cav-1 protein levels in EVs from stretched VSMCs transfected with Cav-1 siRNA versus scramble siRNA (Fig. 3F).

### Actin filament dynamics regulate caveolin-1 trafficking

**Fig. 4:**
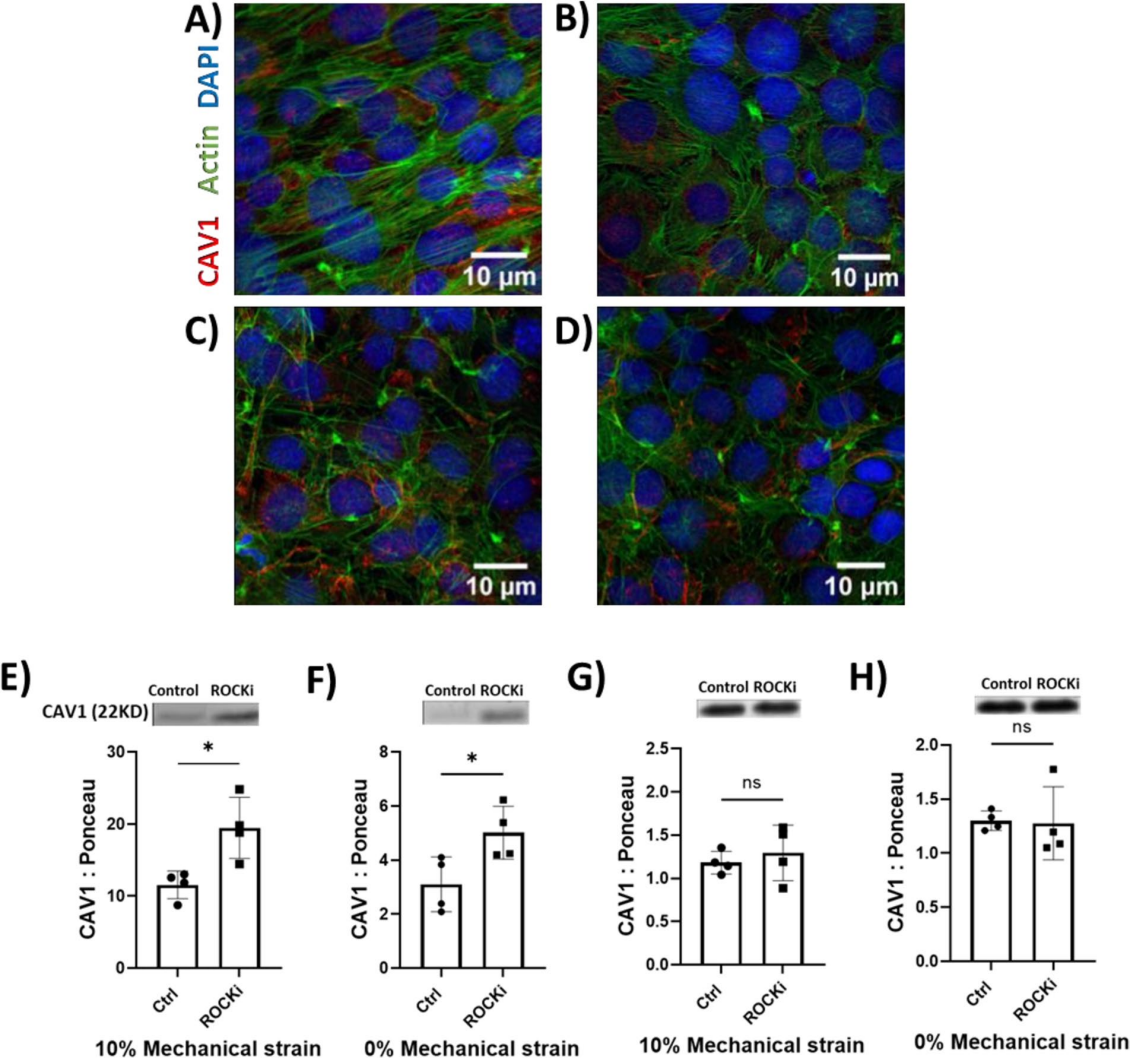
RhoA kinase (ROCK) inhibition increases Cav-1 EVs independently of intracellular Cav-1 levels. Phalloidin staining shows reorientation of F-actin (green) in VSMCs subjected to 10% cyclic strain (**A**) versus non-stretched VSMCs (**B**). ROCK inhibitor Y-27632 (10 μM) disrupts F-actin in both stretched (**C**) and static (**D**) VSMCs. This associates with increased EV Cav-1 release from stretched (**E**) and non-stretched (**F**) VSMCs treated with Y-27632 versus vehicle control. Intracellular Cav-1 levels are unaltered by ROCK inhibition in stretched (**G**) or static (**H**) VSMCs. *n* = 4. Data are mean ± SD. (*P < 0.05). (Scale bar: 10 µm). Data represents mean ± SD. Full-length blots are presented in Supplementary Fig. 4

Exposure of VSMCs to 10% mechanical stretch led to actin stress fiber reorientation in a uniform direction compared to non-stretched cells (Fig. 4A and B) as indicated by phalloidin stain. Recent reports have described the critical role of actin–myosin network in mobilization and translocation of vesicles to the plasma membrane [26]. To investigate the role of actin filament dynamics in stretch-induced Cav-1 trafficking, we inhibited RhoA kinase (ROCK) activity using the small molecule Y-27632 to disrupt actin filament polymerization and contractility (Fig. 4C and D). Interestingly, ROCK inhibition significantly exacerbated Cav-1 EV content in both stretched and non-stretched VSMCs (*P* < 0.05) (Fig. 4E and F). ROCK inhibition did not change the intracellular level of Cav-1 in either group (Fig. 4G and H).

### Mechanical stretch induces mineral potential of liberated EVs

**Fig. 5:**
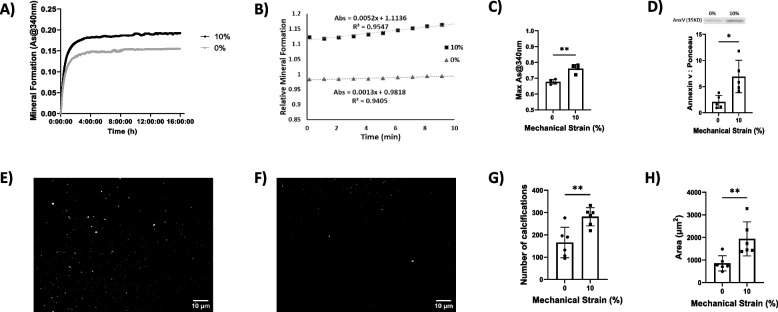
**A**, **B** Light absorbance at 340 nm reveals that EVs from 10% stretched VSMCs form more calcific mineral. **C** Normalization with respect to control samples reveals higher mineralization rate for 10% stretch. **D** Annexin V, a calcium binding membrane protein, in EVs increased in response to increases in mechanical stretch. Collagen hydrogel assay to visualize EV-induced mineralization for stretched cells (**E**) compared to non-stretched control (**F**). **G** Mechanical stretch significantly elevated the number of the mineral nucleation sites. H) Total mineralized area was increased by EVs from mechanically stretched VSMCs. n ≥ 4. Data are mean ± SD (*P < 0.05, **P < 0.01). (Scale bar: 10 µm). Full-length blot is presented in Supplementary Fig. 5

To determine whether mechanical stretch changes the calcifying potential of released EVs, we examined the conditioned media of VSMCs using a mineral formation assay. EVs from VSMCs exposed to 10% mechanical stretch exhibited a 20.9 ± 0.7% increase (*P* < 0.01) in the maximum absorbance of 340 nm light compared to EVs from non-stretched VSMCs (Fig. 5A and B). A linear regression during the rapid phase of mineralization revealed that the absorbance increase occurred fourfold faster in the stretched samples compared to non-stretched samples (Fig. 5C). Annexin V is a calcium-binding membrane protein that helps mediate mineral formation [27]. We observed 3.4-fold (*P* < 0.05) increase in Annexin V content in the isolated EVs of 10% stretched VSMCs compared to non-stretched cells (Fig. 5D).

In order to directly visualize the impacts of mechanical stimulation on EV-mediated mineralization, a 3D collagen hydrogel system was utilized as an in vitro biomimetic calcification model [6]. Quantitative image analysis of the fluorescent micrographs was then carried out using ImageJ software to determine the number and total area of mineralization sites within the hydrogels (Fig. 5E and F). Segmentation and particle analysis revealed a 1.96-fold increase (*P* < 0.01) in the number of mineral nucleation sites formed by EVs derived from mechanically stretched cells compared to non-stretched ones (Fig. 5G). Additionally, total area of the mineralization sites was elevated by 2.2-fold (*P* < 0.01) in hydrogels treated with EVs from mechanically stimulated cells versus non-stretch condition (Fig. 5H).

### Cyclic stretch amplifies the pro-calcific VSMC phenotype induced by osteogenic culture

**Fig. 6:**
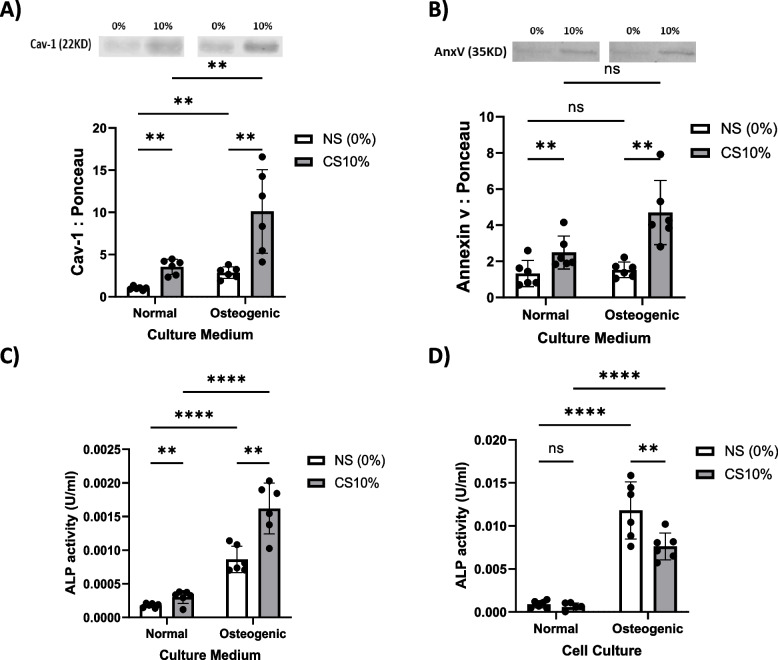
Osteogenic factors and cyclic stretch induce pro-calcific VSMC phenotype and EV release. **A** Osteogenic media increased EV Cav-1 1.85-fold and stretch augmented Cav-1 2.5-fold. **B** Osteogenic conditions elevated EV annexin V 0.88-fold and 0.86-fold with stretch and non-stretch. **C** EV alkaline phosphatase (ALP) rose 5.86-fold and 3.44-fold in osteogenic media with stretch and non-stretch. Stretch also increased EV ALP 0.7-fold in normal media. **D** Cellular ALP increased 12.5-fold and 12.2-fold with osteogenic media and stretch reduced intracellular ALP 0.35-fold and 0.36-fold. *n* = 6. Data are mean ± SD (**P < 0.01, ***P < 0.001, ****P < 0.0001). Full-length blots are presented in Supplementary Fig. 6

Culturing VSMCs in osteogenic media containing dexamethasone, ascorbic acid, and β-glycerophosphate induced a significant 2.85-fold increase (*P* < 0.01) in Cav-1 content in EVs released from the cells versus EVs from VSMCs maintained in normal growth media (Fig. 6A). This is indicative of increased secretion of Cav-1-positive EVs under osteogenic conditions as shown previously [7]. Application of 10% cyclic mechanical stretch further augmented Cav-1-EV release from VSMCs cultured in osteogenic media. Quantitative immunoblotting revealed a significant 3.5-fold increase (*P* < 0.01) in Cav-1 levels in EVs released from stretched VSMCs grown in osteogenic versus normal media (Fig. 6A). Culturing VSMCs in osteogenic media led to increased Annexin V content in EVs released from the cells. Quantitative immunoblotting revealed a significant 1.88-fold increase in Annexin V levels in EVs released from cyclically stretched VSMCs following culture in osteogenic media compared to normal media. A 1.86-fold elevation in Annexin V was also observed in EVs from static VSMCs grown in osteogenic versus normal conditions (Fig. 6B).

Culturing VSMCs in osteogenic media led to a 6.86-fold (*P* < 0.001) and 4.44-fold (*P* < 0.001) increase in tissue non-specific alkaline phosphatase (ALP) activity in the EVs released from cyclically stretched and non-stretched VSMCs, respectively, compared to cells grown in normal media. This demonstrates induction of a pro-calcific phenotype under osteogenic conditions [6]. Interestingly, a significant 1.7-fold increase (*P* < 0.01) in EV ALP activity was also observed in EVs from VSMCs cultured in normal growth media and subjected to cyclic mechanical stretch (Fig. 6C). Within the cells, osteogenic media elicited a significant 13.5-fold and 13.2-fold increase in intracellular ALP activity (*P* < 0.001) compared to normal media for stretched and non-stretched VSMCs, respectively. Application of cyclic mechanical stretch reduced intracellular ALP levels by 1.54-fold and 1.58-fold in osteogenic and normal culture media, respectively (Fig. 6D).

## Discussion

The critical impact of hypertension-induced stress on VSMCs and arterial remodeling—including calcification—has been well described in previous studies [17, 28]. Cav-1, a structural protein in caveolae, is required for the formation of calcifying EVs [7]. Additionally, it has been shown that disassembly of caveolae buffers sudden increases in stress on the plasma membrane [15]. However, the effect of mechanical stimulation on calcifying EV formation in VSMCs has not been reported. To address this knowledge gap, we investigated in vitro behavior of VSMCs in response to mechanical stimulation and showed that mechanical stretch regulates Cav-1 trafficking and its redistribution from VSMCs into EVs. Further, EVs from VSMCs exposed to mechanical stretch have increased mineralization potential.

Lipid rafts are distinct microdomains within the plasma membrane that are enriched in sphingolipids and cholesterol [29, 30]. This unique lipid composition renders lipid rafts and caveolae less fluid and more tightly packed than the surrounding phospholipid bilayer. While both planar lipid rafts and caveolae domains are involved in signal transduction, planar lipid rafts form small, transient platforms that float freely within the membrane. In contrast, caveolae are invaginated flask-shaped structures that are stabilized by the structural protein caveolin. The high cholesterol content of caveolae allows them to function as organized signaling centers that compartmentalize and regulate signaling molecules. The cyclic strain-induced translocation of Cav-1 from caveolae to non-caveolar domains observed in our study may have significant functional consequences by modulating Cav-1-mediated cell signaling. Upon caveolae disassembly and Cav-1 displacement into other membrane regions, dissociation of Cav-1 from signaling effectors can then lead to changes in their activation state. For example, previous studies have shown that mechanical stimuli trigger caveolae disassembly and activation of Src family kinases and ERK pathway in a mechanism dependent on Cav-1 redistribution [25, 31]. Our data also suggest that the application of mechanical stretch increases total intracellular cholesterol after 24 h, perhaps reflecting a feedback attempt to recover plasma membrane homeostasis following the sudden addition of cyclic tension. Further investigation into the specific signaling pathways affected by Cav-1 displacement from caveolae will provide insights into mechanisms of vascular mechanotransduction.

Our results demonstrate that cyclic mechanical stretch alters Cav-1 distribution in VSMCs compared to non-stretched conditions, reducing intracellular Cav-1 while increasing secretion into EVs. Additionally, stretch increased intracellular cholesterol in VSMCs. This cholesterol accumulation may represent a compensatory response to restore caveolae domains disrupted by plasma membrane deformation. Further investigation into how forces stimulate caveolin-1 trafficking and cholesterol biosynthesis, as well as mobilization into EVs using diverse in vitro and ex vivo stretch protocols, will provide insights into the coordination of these processes. Understanding the links between strain-sensitive caveolin-1 redistribution, cholesterol metabolism, and secretion may reveal fundamental mechanisms mediating intercellular communication networks in response to vascular biomechanics.

Previous studies suggested that caveolae endocytosis occurs in response to plasma membrane damage [32, 33]. Our observation of Cav-1 re-localization from the plasma membrane to the cytosol in VSMCs exposed to cyclic mechanical stretch is consistent with caveolae endocytosis induced by membrane strain. VSMCs exposed to cyclic mechanical stretch do not exhibit reduced viability compared to non-stretched samples (Supplemental Fig. 1), suggesting that the observed increase in extracellular Cav-1 is due to active trafficking within live cells. As noted above, density gradient fractionation of stretched VSMCs revealed a shift of Cav-1 out of buoyant, caveolae-enriched fractions into denser, non-caveolar lipid domains. This implies that caveolae may internalize into the cytosolic space at these domains upon mechanical perturbation. Dynamin has been identified as a key mediator of caveolae budding and endocytosis [34, 35]. Accordingly, dynamin inhibition reduced Cav-1 release in EVs from stretched VSMCs, implicating caveolae internalization as a prerequisite for loading Cav-1 into EVs with cyclic strain. The exosomal marker CD63 was unchanged in EVs from stretched versus non-stretched VSMCs, indicating distinct regulation of Cav1-derived vesicles. Cav-1 has been previously associated with regulation of microRNA sorting into microvesicles [36], which bud directly from the plasma membrane of cells. Future studies in intracellular trafficking and exosome-like secretion of Cav-1-positive EVs may yield new insight into the regulation of this unique EV population.

The actin cytoskeleton often mediates cellular responses to mechanical stimulation [37]. Besides the regulation of membrane tension, actin stress fibers directly associate with caveolae and play a critical role in the intracellular trafficking, endocytosis and exostosis of vesicles [14, 38]. Rho-A kinase (ROCK) is a member of the GTPase family and participates in actin filament polymerization and contraction [39]. Additionally, ROCK contains a Cav-1-binding domain [40]. ROCK inhibition reduced actin polymerization in VSMCs, yet increased Cav-1 release in extracellular vesicles. These results reveal that actin dynamics influence Cav-1 externalization, though the mechanisms remain unclear. Ongoing studies are needed to delineate how alterations in actin polymerization and contractility modulate EV Cav-1 loading, including the potential contributions of actin enhancement pathways. Elucidating the cytoskeletal control of Cav-1 trafficking will provide broader insights into how the actin network integrates mechanical cues to direct intercellular communication through extracellular vesicles.

Vascular calcification often associates with hyperphosphatemia [41]. Our results revealed that EVs released from stretched VSMCs have higher potential to form calcium-phosphate mineral when they are placed in a high phosphate environment. Annexin V is a phospholipid-binding protein that has previously been implicated in mineral nucleation within EVs [27]. We detected an increase in annexin V protein in EVs from VSMCs subjected to 10% strain. The 3D collagen hydrogel platform allowed us to visualize mineral nucleation as an in vitro model of vascular calcification. Quantitative image analysis revealed increased numbers of mineral nucleation sites and total area covered by mineral in hydrogels treated with EVs from cyclically stretched VSMCs. These biomimetic calcification assays confirm that mechanical forces enhance the release of EVs with greater propensity to induce calcium phosphate deposition, complementing our cell culture data. Together, these data suggest that mechanical stimulation leads to release of EVs with elevated mineralization potential. Future studies will directly assess the causal role of altered Cav-1 trafficking in promoting the increased mineralization potential.

## Conclusion

**Fig. 7:**
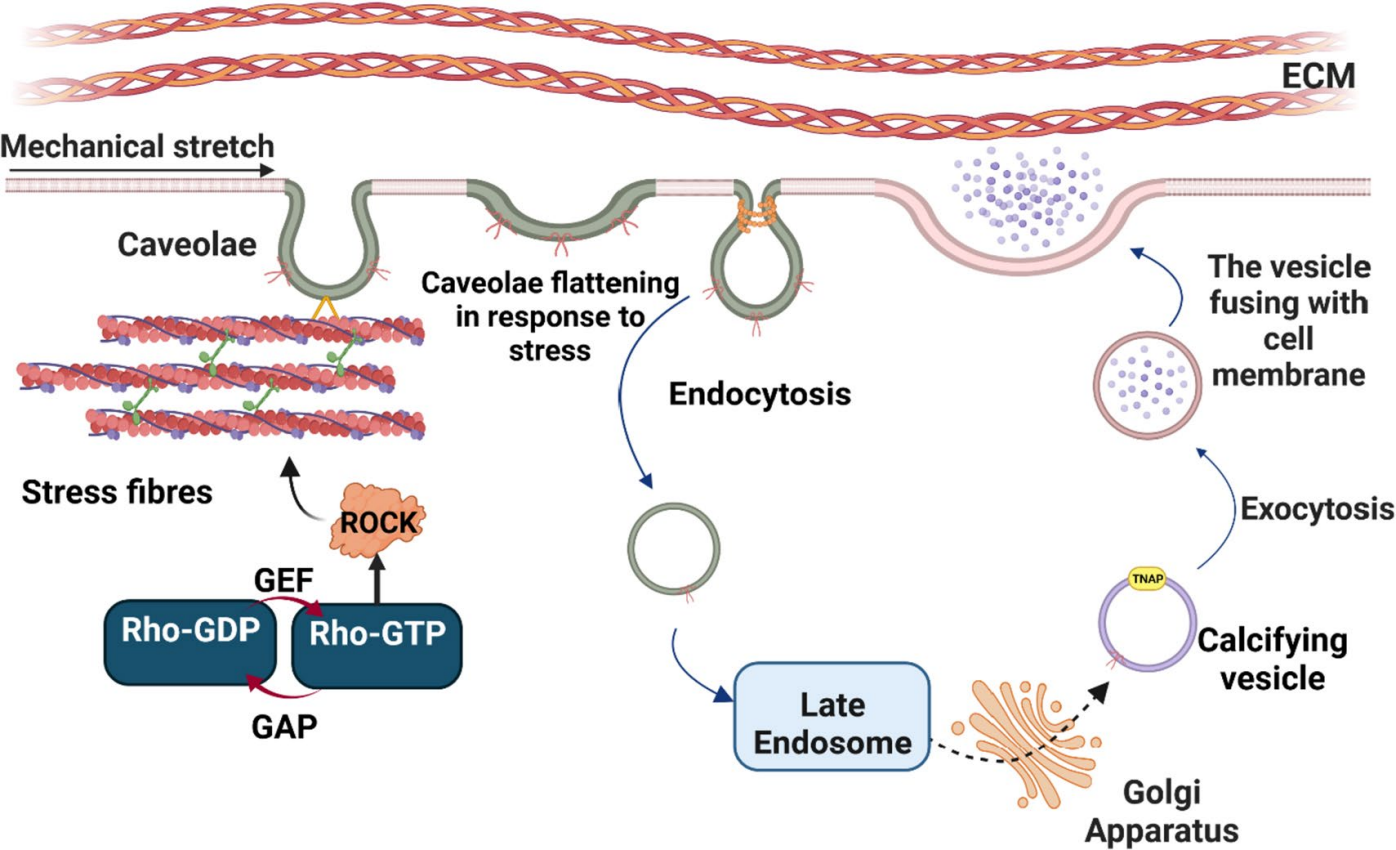
Proposed mechanism of cyclic mechanical stretch-induced caveolin-1 trafficking in vascular smooth muscle cells. Mechanical stimulation promotes caveolae endocytosis in a dynamin-dependent process, reducing caveolin-1 levels in the plasma membrane. Caveolin-1 is subsequently trafficked to the cytosol and incorporated into extracellular vesicles, leading to increased release. The extracellular vesicles from stretched cells exhibit increased alkaline phosphatase (ALP) activity. RhoA kinase (ROCK) mediates actin cytoskeleton remodeling in response to stretch, which regulates caveolin-1 mobilization

In conclusion, this study shows that cyclic stretch alters Cav-1 distribution and enhances release in EVs from VSMCs. Stretch-induced caveolin-1 mobilization involves caveolae endocytosis. Moreover, EVs from stretched VSMCs exhibit increased mineralization potential. A proposed mechanism diagram revealing the regulation of cyclic mechanical stretch on Cav-1 redistribution and extracellular vesicle release in vascular smooth muscle cells is shown in Fig. 7. Further research on links between biomechanics, Cav-1 dynamics, and EV function will provide insights into vascular disease progression.

## Materials and methods

### Cell culture and mechanical stretch

Primary mouse and human VSMCs (ATCC, Manassas, VA) were cultured in Dulbecco’s Modified Eagle’s Medium (DMEM) supplemented with 10% fetal bovine serum and 1% penicillin–streptomycin at 37 °C in a humidified atmosphere of 5% CO2 and 95% air. After reaching 80–90% confluence, VSMCs between passages 3–8 were used for experiments. In addition to mouse vascular smooth muscle cells, a human cell line (ATCC, Manassas, VA) was utilized for RNA interference and osteogenic differentiation experiments in order to translate and validate key findings across species. To apply mechanical stretch, VSMCs with the cell density of 2 × 10^5^ cells/well were seeded onto collagen type I pre-coated 6-well Bioflex® plates (Flexcell International Crop, Burlington, NC) in the presence of DMEM, 1% penicillin/streptomycin and 10% EVs deprived serum. EV-deprived serum was obtained by ultracentrifugation at 100,000 × g for 15 h, after which the supernatant was collected for use. To apply 10% equibiaxial mechanical stretch (0.5 Hz, 72 h) on cultured VSMCs, a Flexcell® FX-5000 T tension system (Flexcell International Corp.) was utilized. Non-stretched VSMCs seeded on 0.2% gelatin-coated tissue culture plastic plates were used to establish a common baseline for comparison between the groups exposed to different levels of stretch. To determine the viability of VSMCs following prescribed culture regimen, we used a commercially available assay kit (Abcam, Cambridge, United Kingdom). The live cell dye penetrates intact, live cells and produces a fluorescence signal (Ex/Em = 485/530 nm) following hydrolysis by active intracellular esterases. The fluorescent dead cell dye (Ex/Em = 495/635 nm) is impermeable to live cells but enters damaged cells and binds to nucleic acids. Following the addition of the dyes according to the manufacturer’s instructions, cells were imaged with a Zeiss Axioscope upright fluorescence microscope. Live and dead cells were counted using ImageJ and used to calculate overall viability.

The following specific inhibitors were used for the prescribed treatments: Dynasore (40 µM; APExBIO, Houston, TX) as GTPase inhibitor of dynamin, and Y-27632 dihydrochloride (10 µM; Biotechne, Bristol, United Kingdom) for RhoA kinase inhibition.

### Osteogenic stimulation

In order to induce a pro-calcific phenotype, VSMCs were cultured in osteogenic media for 7 days prior to applying 10% mechanical stretch, in order to induce a pro-calcific phenotype. The osteogenic media consisted of complete control media supplemented with dexamethasone (10 nM; Sigma), L-ascorbic acid (100 μM; Sigma), and β-glycerolphosphate (10 mM; Sigma).

### Western blot analysis

VSMCs lysates were prepared in ice-cold RIPA Buffer. EVs were isolated from the conditioned media of VSMCs via centrifugation at 1000 g for 5 min to remove cell debris followed by ultracentrifugation at 100,000 g for 60 min at 4 °C using an Optima Max-TL ultracentrifuge (Beckman Coulter, Brea, CA). Isolated EVs were lysed in protein lysis buffer. Following the BCA protein concentration assessment, equal amounts of the lysates were separated with 10% SDS–polyacrylamide gel electrophoresis and then transferred onto nitrocellulose membranes (Bio-Rad, Hercules, CA). Due to lack of appropriate loading control for the EVs, Ponceau S was used to confirm equal protein loading. Membranes were blocked for 1 h at room temperature in casein blocking buffer in TBST (Sigma-Aldrich, St. Louis, MO) and then 1–2 h at room temperature with primary antibodies for Caveolin-1 (1:500; Abcam, Cambridge, United Kingdom), Annexin V (1:500; Abcam, Cambridge, United Kingdom) or CD63 (1:500; Abcam, Cambridge, United Kingdom). Following washing, the species appropriate secondary antibody (1:10,000; LI-COR, Lincoln, NE) was added for 1 h at room temperature. Visualization was performed using an Odyssey®CLx (LI-COR, Lincoln, NE) and band densities were analyzed using Image Studio® (LI-COR).

### Caveolae/lipid raft isolation

Isolation of detergent-resistant membranes enriched in caveolae and lipid rafts was performed using a caveolae/raft isolation kit (Sigma-Aldrich) according to the manufacturer's instructions. Briefly, cultured VSMCs were lysed in ice-cold lysis buffer containing 1% Triton X-100 and homogenized. The lysates were combined with an equal volume of 60% OptiPrep density gradient medium and overlaid with layers of 35% and 5% OptiPrep in gradient buffer. The samples were ultracentrifuged at 200,000 × g for 4 h at 4 °C to allow separation of detergent-soluble and insoluble fractions across the gradient. Detergent-resistant membranes enriched in caveolae/raft components floated to the 5–35% interface (1–7 fractions) and were collected. These buoyant membrane fractions were confirmed to be enriched in canonical lipid raft markers by immunoblotting of Cav-1.

### Cellular cholesterol measurment

Cellular lipids and cholesterol were extracted from cultured VSMCs using an organic solvent-based method. Briefly, VSMCs were homogenized in a 3:2 hexane:isopropanol solution to lyse cell membranes and extract lipids. The homogenates were centrifuged to separate the aqueous and organic phases. The upper organic phase containing lipids was transferred to a clean microcentrifuge tube and dried under vacuum to recover total cellular lipids. Immediately prior to quantification, the dried lipids were resuspended in isopropanol. Total cholesterol content was measured employing Cholesterol E kit (Fujifilm Wako Diagnostics) enzymatic colorimetric assay kit according to the manufacturer's protocol.

### Immunofluorescence analysis

VSMCs were fixed with 4% formalin for 10–15 min and permeabilized by 0.1% Triton X-100 (Fisher scientific, Waltham, MA) for 10 min. Non-specific binding sites were blocked with 0.1 mM glycine in 1% BSA for 30 min at room temperature. Next, cells were incubated with caveolin-1 primary antibody (1:200) for 2 h at room temperature, washed with 1X PBS, and incubated with secondary antibody Alexa Fluor® 594 (1:400; Abcam, Cambridge, United Kingdom) for 1 h at room temperature. Cells were co-stained with Phalloidin-iFluor™ 488 Conjugate (1:1000; Cayman chemical, Ann Arbor, MI) for 30 min. The stained cells were mounted in Permount™ Mounting Medium (Fisher scientific) and imaged using a laser scanning confocal microscope (Eclipse Ti, Nikon, Minato City, Tokyo, Japan).

### RNA interference

To silence caveolin-1 expression, 50 nm siRNA against caveolin-1 (s2448, Life Technology) and non-targeting siRNA (ON-TARGET Non-Targeting Pool, Thermo Scientific) was transfected into VSMCs using (Thermo Scientific). Transfection was performed 48 h prior to mechanical stretch exposure.

### Mineral formation assay

In order to measure the real-time mineral formation of EVs, we used an assay originally developed to study mineral formation from growth plate cartilage EVs [42]. For kinetic assay, 1% (v/v) of 300 mM NaH_2_PO_4_ (Sigma-Aldrich, St. Louis, MO) was added to 200 µl of EVs stock solution of each sample and suspensions were placed into 96 well plates and incubated at 37 °C in a Synergy HT microplate reader (Biotek, Winooski, VT) for 16 h. The absorbency reading was recorded at 340 nm at 1-min interval.

### Collagen hydrogel experiments

Collagen hydrogels were prepared by mixing high-concentration rat tail collagen with DMEM in a 1:19 ratio and neutralizing the mixture to pH 7–8. The neutralized collagen solution was then added to chambered coverglass wells (LAB-TEK). The conditioned media of VSMCs, containing EVs, were added to the collagen network and incubated at 37 °C for the indicated time period. One day before imaging, a near-infrared-based bisphosphonate calcium tracer (OsteoSense 680, Perkin Elmer) was added to the hydrogels and incubated overnight at 37 °C. The resulting aggregation and calcification processes were imaged by fluorescence microscopy (Ziess AXIO). Quantitative image analysis of the captured fluorescence micrographs was performed using ImageJ software (NIH).

### Alkaline phosphatase activity assay

Alkaline phosphatase (ALP) activity in cells and EVs was measured using a colorimetric assay kit (Abcam) according to the manufacturer’s protocol. Briefly, cell lysates were collected in the supplied buffer and centrifuged to remove insoluble material. The supernatants were added to a 96-well plate at a volume of 80 μL per well after dilution with the provided assay buffer. Diluted p-nitrophenyl phosphate (pNPP) substrate was then added to each sample well and the plate was incubated at 25 °C protected from light for 1 h to allow the enzymatic reaction to occur. The reaction was stopped by addition of a stop solution. Finally, the absorbance was measured at 405 nm using a microplate reader to quantify ALP activity.

### Statistical analysis

Each experiment was performed for at least three biological replications, and all data were presented as mean ± SD. Student’s t-test was used to analyze two group comparisons. Multiple group comparisons were conducted by one-way ANOVA. All statistical tests were calculated using GraphPad Prism 9.00 (GraphPad Software). A *P*-value less than 0.05 was considered statistically significant.

### Supplementary Information

**Supplementary Material 1.**

## Data Availability

The complete raw datasets employed and/or examined in this study will be made available upon reasonable request to the corresponding author.

## Abbreviations

ALP: Alkaline phosphatase
AnxV: Annexin V
Cav-1: Caveolin 1
EV: Extracellular vesicle
ROCK: RhoA Kinase
VSMCs: Vascular smooth muscle cells
