# Supplementary material for: Mechanical stretch leads to increased caveolin-1 content and mineralization potential in extracellular vesicles from vascular smooth muscle cells
Source: BMC Mol Cell Biol. 2024 Mar 14;25:8. doi: 10.1186/s12860-024-00504-w (PMC10938675; doi:10.1186/s12860-024-00504-w)
Supplement: Supplementary file 1 — Supplementary Material 1. [file 12860_2024_504_MOESM1_ESM.pdf]

Supplemental Data

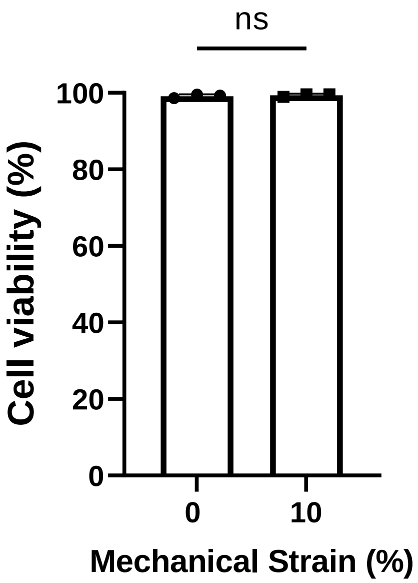

Supplemental Fig. 1. Cell viability analyses show no difference between VSMCs exposed to 10% cyclic stretch and non-stretched VSMCs (n=3).

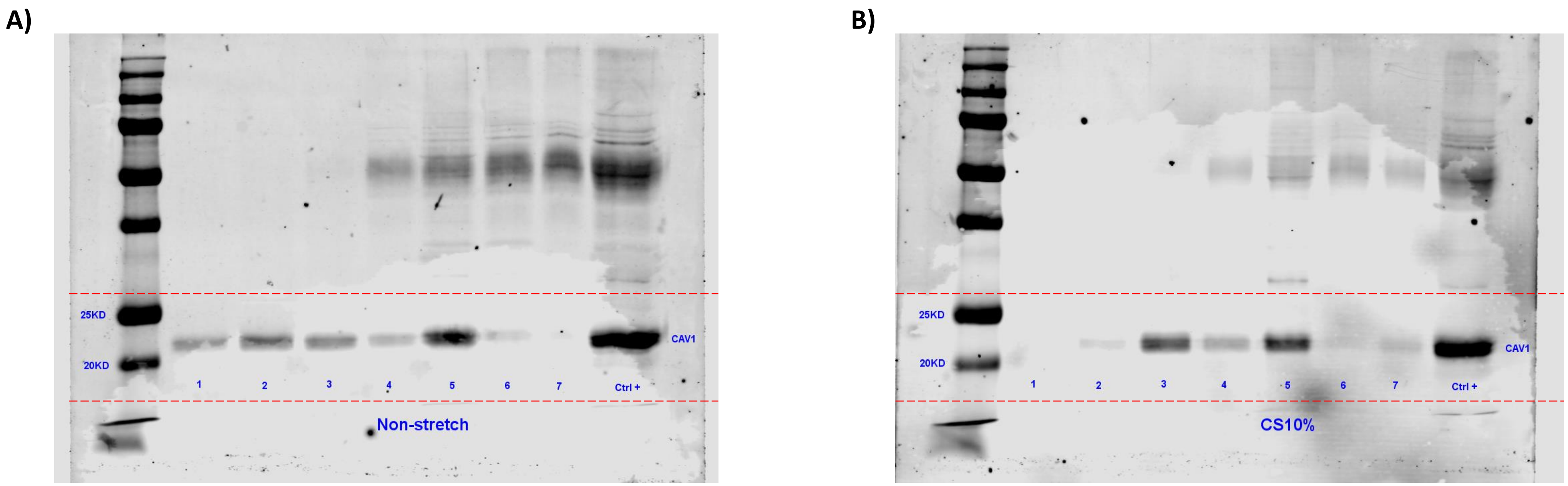

Supplementary Fig. 2. Caveolin-1 blot of density graduate centrifugation. (A) non-stretch VSMCs (B) 10% stretched VSMC. In the image, the portion of the membrane used for analysis is outlined by dashed lines.

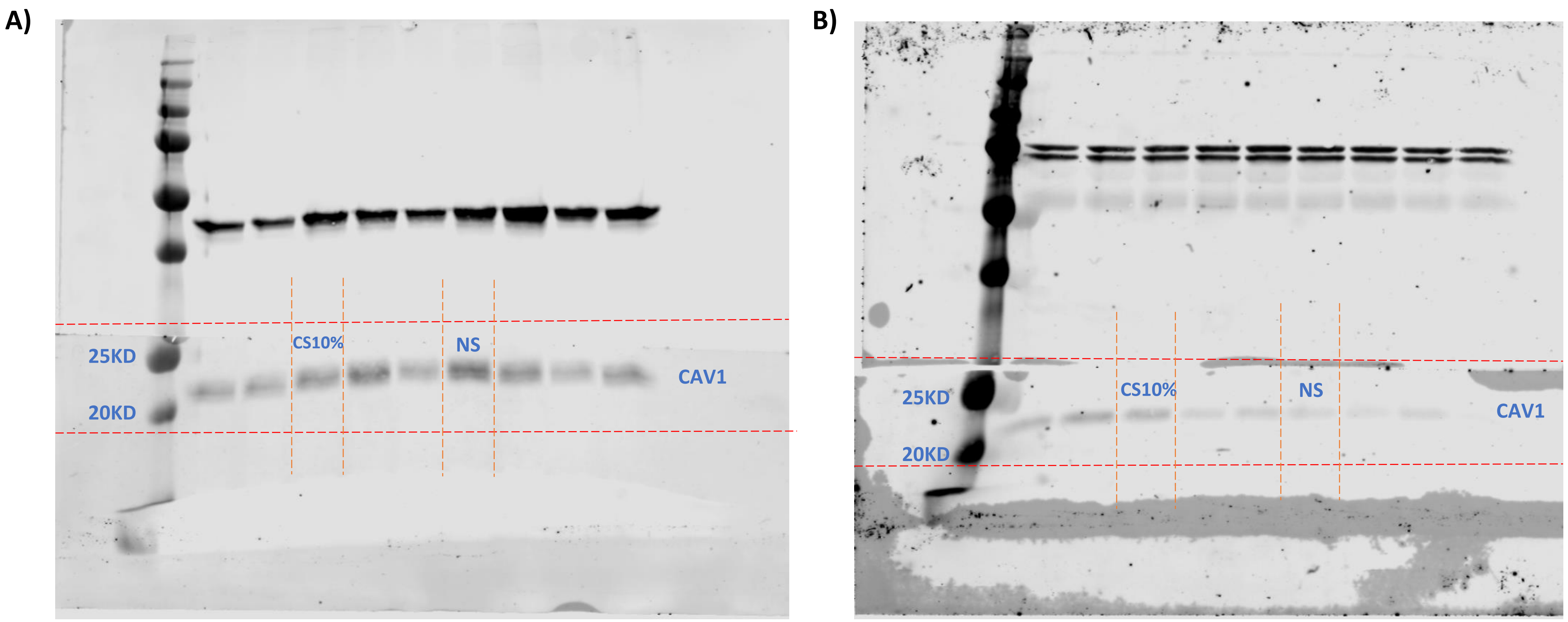

Supplementary Fig. 3. Caveolin-1 blotting of 10% stretch (CS10%) and non-stretch (NS) VMSCs . (A) Intercellular (B) Extracellular. In the image, the portion of the membrane used for analysis is outlined by dashed lines.

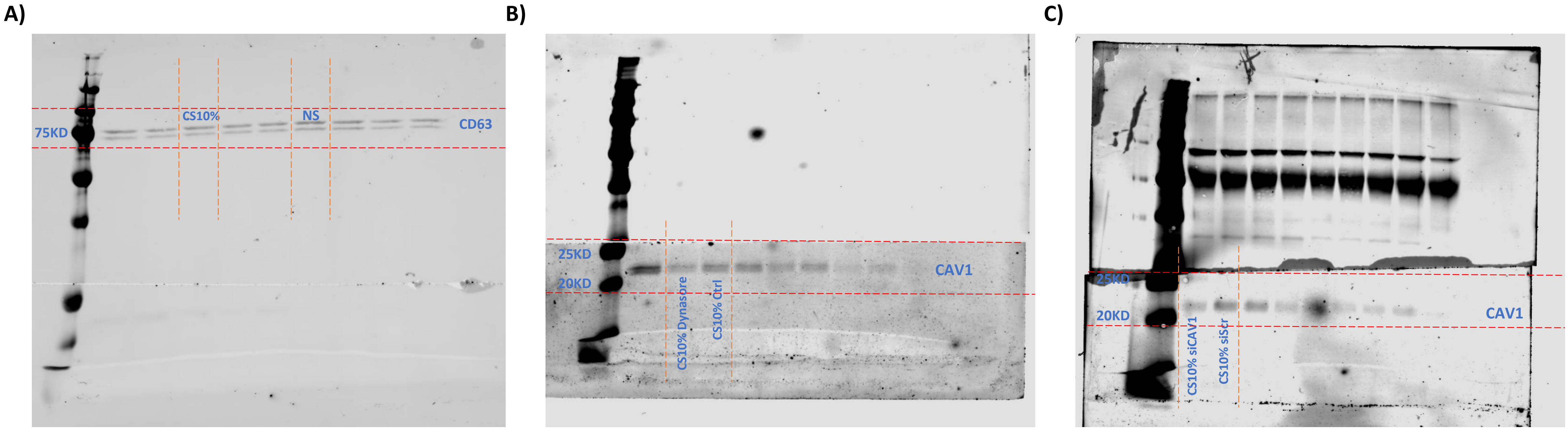

Supplementary Fig. 4. (A) CD63 blotting of extracellular vesicles (EVs) isolated from VSMCs subjected to 10% cyclic stretch (CS10%) and non-stretched conditions (NS). (B) Caveolin-1 blotting of isolated EVs from Dynasore-treated and untreated control VSMCs exposed to 10% mechanical stretch. (C) Caveolin-1 blotting of isolated EVs from CAV1 gene knockdown (siCAV1) and scramble control (siScr) VSMCs subjected to 10% cyclic stretch. In the image, the portion of the membrane used for analysis is outlined by dashed lines.

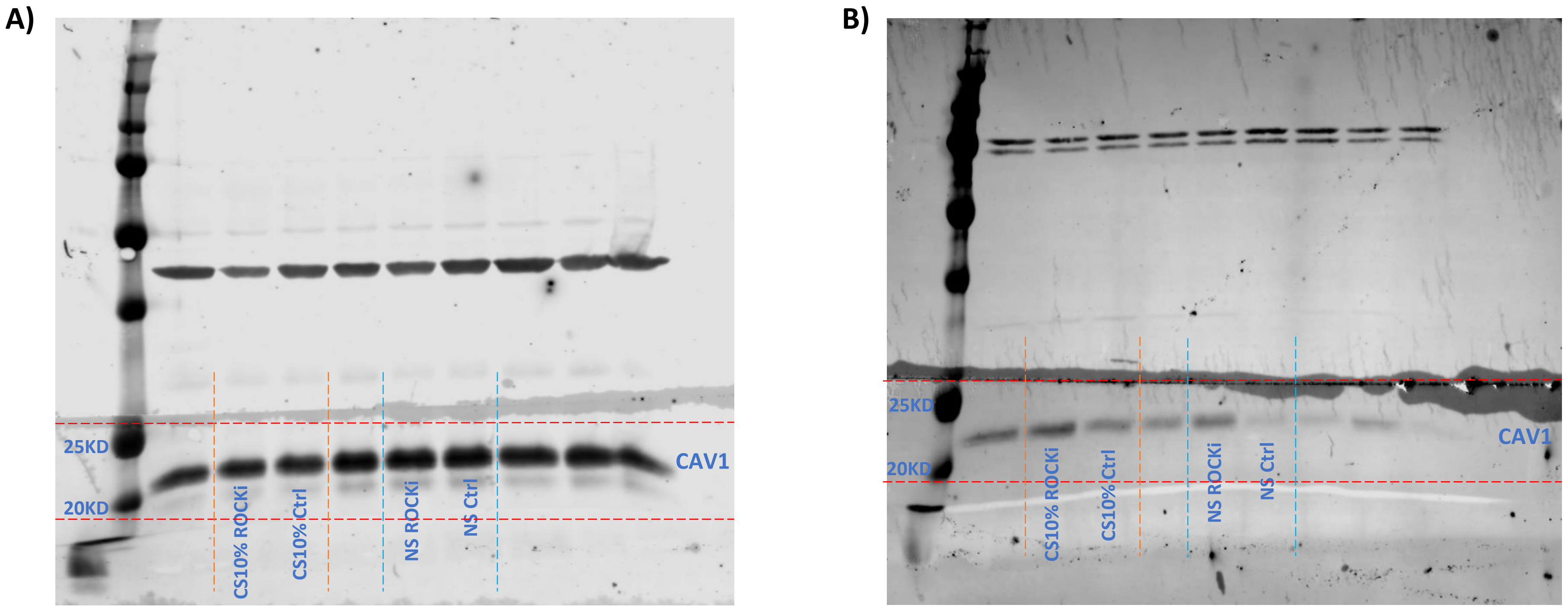

Supplementary Fig. 5. Caveolin-1 blot of RohA kinase inhibitor (ROCKi)-treated and untreated control VSMCs under 10% cyclic stretch (CS10%) and non-stretched (NS) conditions. (A) Intracellular, (B) Extracellular. In the image, the portion of the membrane used for analysis is outlined by dashed lines.

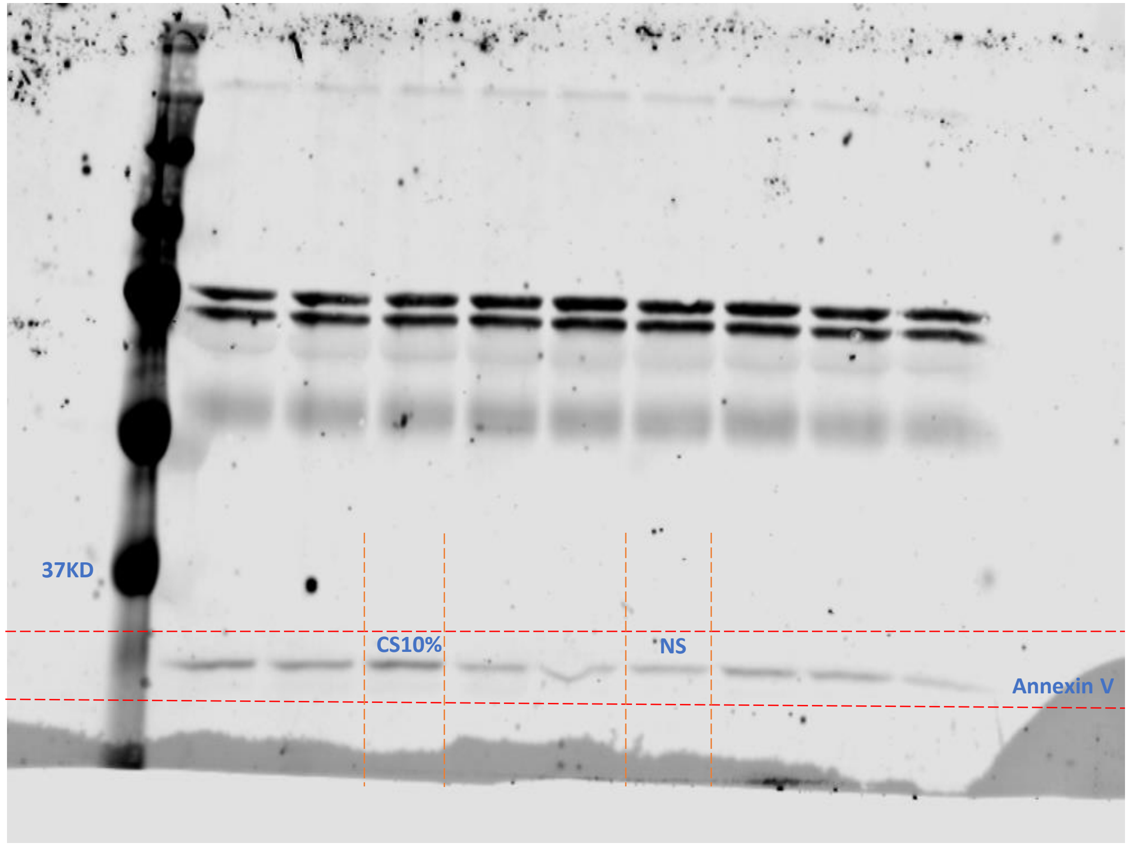

Supplementary Fig. 6. Annexin V blotting of extracellular vesicles (EVs) isolated from VSMCs under 10% cyclic stretch (CS10%) and non-stretched (NS) conditions. In the image, the portion of the membrane used for analysis is outlined by dashed lines.

A)

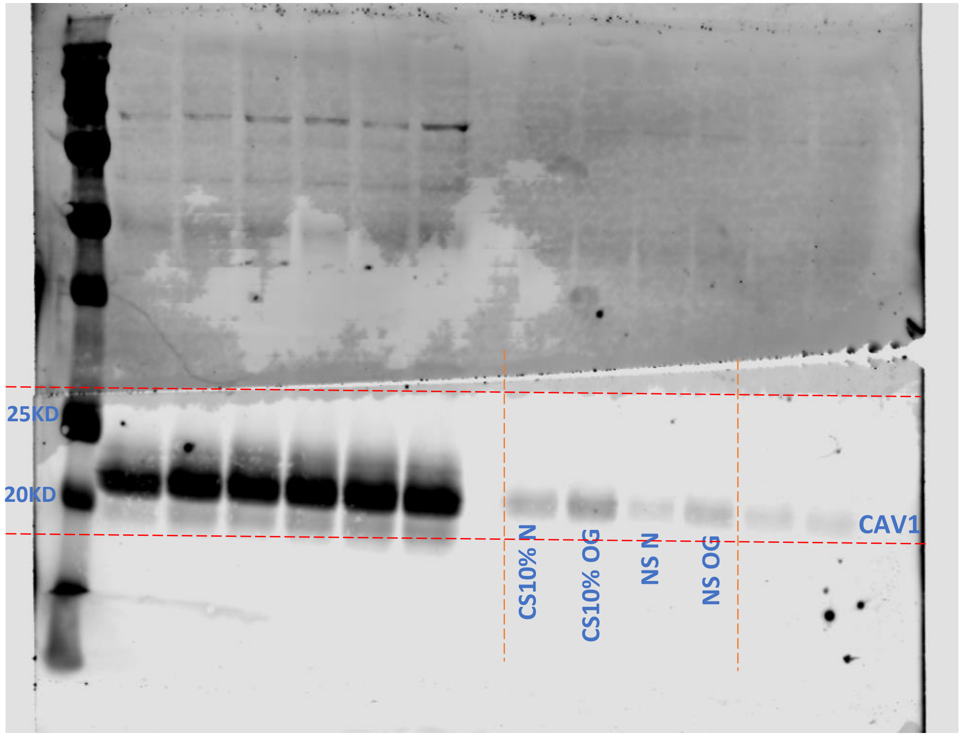

B)

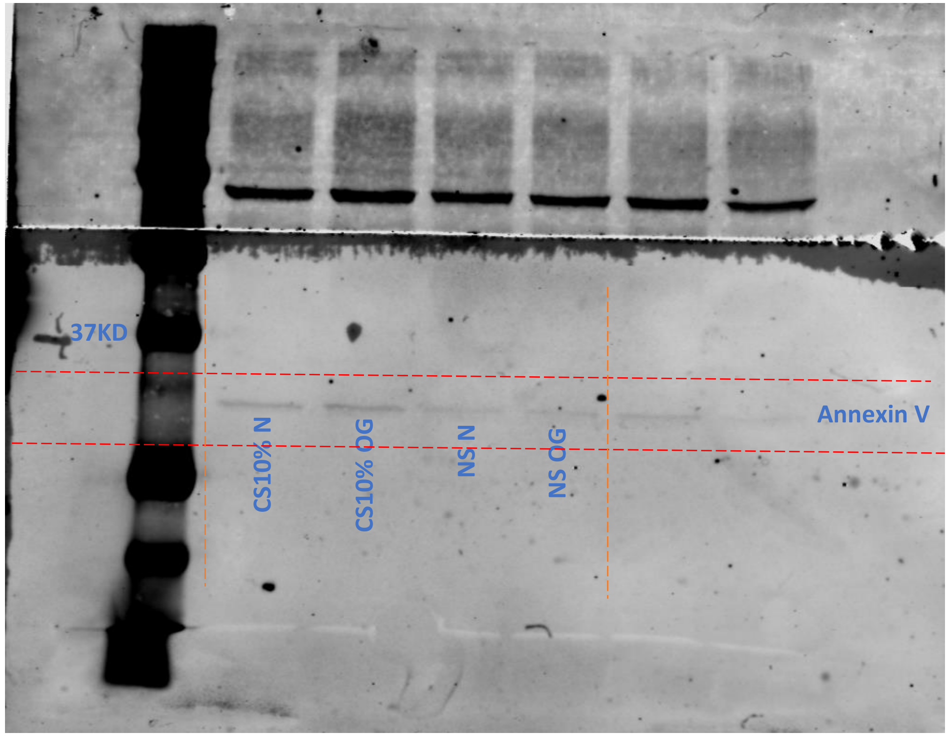

Supplementary Fig. 7. (A) Caveolin-1 blotting of VSMCs cultured in normal (N) and osteogenic (OG) media under 10% cyclic stretch (CS10%) and non-stretched (NS) conditions. (B) Annexin V blotting of VSMCs cultured in normal (N) and osteogenic (OG) media under 10% cyclic stretch (CS10%) and non-stretched (NS) conditions. In the image, the portion of the membrane used for analysis is outlined by dashed lines.
